# Oligonucleotides Targeting Telomeres and Telomerase in Cancer

**DOI:** 10.3390/molecules23092267

**Published:** 2018-09-05

**Authors:** Zachary Schrank, Nabiha Khan, Chike Osude, Sanjana Singh, Rachel J. Miller, Collin Merrick, Alexander Mabel, Adijan Kuckovic, Neelu Puri

**Affiliations:** Department of Biomedical Sciences, University of Illinois College of Medicine at Rockford, Rockford, IL 61107, USA; zacharyschrank15@augustana.edu (Z.S.); nkhan28@uic.edu (N.K.); cosude2@uic.edu (C.O.); ssingh92@uic.edu (S.S.); rjmille2@uic.edu (R.J.M.); 1998cem@gmail.com (C.M.); alexander.mabel2001@gmail.com (A.M.); akucko2@uic.edu (A.K.)

**Keywords:** T-oligo, GRN163L, miRNA, imetelstat, telomere, telomerase

## Abstract

Telomeres and telomerase have become attractive targets for the development of anticancer therapeutics due to their involvement in cancer cell immortality. Currently, several therapeutics have been developed that directly target telomerase and telomeres, such as telomerase inhibitors and G-quadruplex stabilizing ligands. Telomere-specific oligonucleotides that reduce telomerase activity and disrupt telomere architecture are also in development as novel anticancer therapeutics. Specifically, GRN163L and T-oligos have demonstrated promising anticancer activity in multiple cancers types via induction of potent DNA damage responses. Currently, several miRNAs have been implicated in the regulation of telomerase activity and may prove to be valuable targets in the development of novel therapies by reducing expression of telomerase subunits. Targeting miRNAs that are known to increase expression of telomerase subunits may be another strategy to reduce carcinogenesis. This review aims to provide a comprehensive understanding of current oligonucleotide-based anticancer therapies that target telomeres and telomerase. These studies may help design novel therapeutic approaches to overcome the challenges of oligonucleotide therapy in a clinical setting.

## 1. Introduction

Traditional cancer treatment modalities use chemotherapy and radiotherapy; however, these treatments often have deleterious side-effects due to toxicities to normal cells. Recent progress has been made in the treatment of cancer using therapeutics that target the molecular mechanisms that control cancer cell survival, proliferation, and invasiveness; these are often termed molecularly-targeted therapies. The use of oligonucleotides against these molecular mechanisms described above has shown therapeutic efficacy against various cancer types in several studies [1,2,3], and thus, oligonucleotides are becoming attractive targeted therapies to improve cancer patient prognosis. 

One attractive application of ribo- and deoxyribo-oligonucleotides as cancer therapeutics is the targeting of telomeres and telomerase activity in cancer cells. Telomerase has garnered interest as a therapeutic target due to its near-universal overexpression in cancer, thus conferring replicative immortality [4]. One such telomerase-targeting therapy is GRN163L (Imetelstat), a 13-mer deoxyribo-oligonucleotide that inhibits telomerase activity by serving as a direct antagonist to the telomerase RNA template hTR [5]. Imetelstat is the most clinically used telomerase inhibitor and has been studied in bladder, breast, liver, prostate, and pancreatic cancers [6]. T-oligos, guanine-rich deoxyribo-oligonucleotides homologous to the 3′-telomeric overhang, are also promising oligonucleotides that have demonstrated anticancer activity in several cancer cell lines [4,7,8]. MicroRNA (miRNA or MiR) are a diverse class of endogenous oligonucleotides that regulate gene expression through RNA interference (RNAi) by interaction with the RNA-Induced Silencing Complex (RISC) and the subsequent degradation of complementary mRNA. Several miRNA have been shown to directly regulate the expression of hTERT, the gene encoding the catalytic subunit of telomerase, thus regulating proliferative activity and evasion of senescence [9,10]. The conservation of TERT is expressed in the functional binding site of miR-128, although miR-138 is conserved between vertebrates, and, in many biological systems, it is not shown to be conserved in the hTERT gene itself [9,11].

Several of these oligonucleotide-based therapies described above have been rigorously studied to elucidate their effects on a multitude of cancer types [12,13]. Novel therapies employing oligonucleotides serve as a promising avenue to overcome the toxic side-effects of conventional cancer therapies. A comprehensive understanding of the mechanism of action of these oligonucleotides and their potential applications is critical for the development of novel therapies. This review aims to examine the role of oligonucleotides that specifically target telomeres and telomerase as novel anticancer therapeutics.

## 2. GRN163L

**Figure 1:**
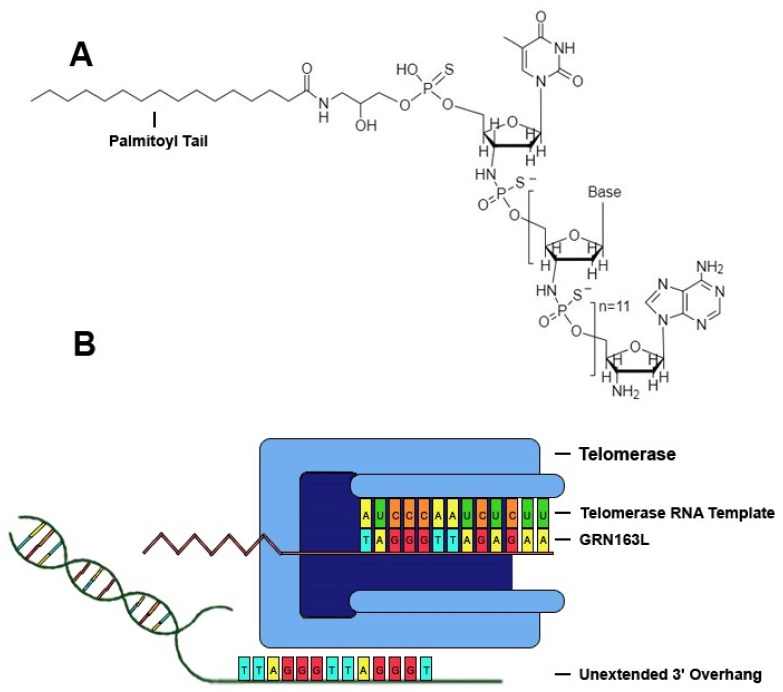
GRN163L inhibits telomerase by binding to hTR. (**A**) Chemical structure of GRN13L. GRN163L is a 13-mer oligonucleotide that base pairs to the RNA template of telomerase. The palmitoyl group bound to the 5′-thio-phosphate group enhances cellular uptake and retention. (**B**) GRN163L base pairs to the RNA template of telomerase, preventing elongation of the 3′ overhang.

Telomerase, a ribonucleoprotein reverse transcriptase that catalyzes the addition of tandem repeats to the 3′ overhang of chromosomes, is comprised of a catalytic subunit with reverse transcriptase activity encoded by the human telomerase reverse transcriptase (hTERT) gene, a RNA template encoded by the human telomerase RNA gene (hTR), and other related proteins, such as dyskerin, NHP2, NOP10 and GAR1 [14]. One of the most promising drugs that specifically targets telomerase is the sodium salt of imetelstat (GRN163L), a synthetic lipid-conjugated 13-mer N3′→P5′ thio-phosphoramidate deoxyribo-oligonucleotide that blocks the template region of telomerase and has potential antineoplastic activity (Figure 1). The anticancer properties of GRN163L have been studied rigorously, and it is the most clinically-studied telomerase inhibitor to date [15,16,17]. GRN163L demonstrates multiple characteristics that prove beneficial in clinical applications, such as high solubility in aqueous solutions, nuclease resistance, and high stability in acidic solutions or the presence of metabolites [14,18]. GRN163L contains a palmitoyl group bound to the 5′-thio-phospate group, which confers hydrophobicity that improves uptake and retention of the drug within biological membranes, and thus enhances telomerase inhibition without the need for transfection [14]. GRN163L contains a complementary sequence to human telomerase RNA (hTR) (5′-palmitate-TAGGGTTAGACAA-NH_2_-3′), allowing it to antagonize a 13-mer sequence of the RNA template of telomerase near the active site, thus conferring inhibition of the enzyme. Inhibition of telomerase activity in cancer cells by GRN163L results in progressive shortening of telomeres and eventual senescence or apoptosis [14].

GRN163L has demonstrated significant inhibition of telomerase in multiple cancer cell lines, resulting in reduction in cell proliferation and lifespan [19,20]. Several pancreatic cancer cell lines treated with GRN163L showed a significant, dose-dependent reduction in telomerase activity independent of basal levels of telomerase activity. Pancreatic cancer cells treated with GRN163L demonstrated similar growth to untreated cells for the first 3–8 weeks, but thereafter began to undergo progressive senescence and apoptosis [19]. Additionally, cells treated with GRN163L exhibited upregulation of γH2AX, a marker of DNA damage response activation. Cells experienced reduction in telomere length for the first five weeks of treatment, but telomere length stabilized upon decline in proliferation, possibly due to the inability of the telomere binding proteins (i.e., the shelterin complex) to block telomerase upon critical telomere shortening [19]. Another study using myeloma cell lines also saw significant inhibition of telomerase activity when treated with GRN1613L, as well as a decline in live cell numbers to <5% of the initial levels over a period of 3–5 weeks [20]. A study using malignant rhabdoid tumor cells evaluated the efficacy of GRN163L both in vitro and in vivo. Activation of DNA damage responses was observed in malignant rhabdoid tumor cells treated with GRN163L as demonstrated by γH2AX foci formation, phosphorylation of ATM, and phosphorylation of TP53. Additionally, in mouse xenograft tumor models, there was a 40–50% reduction in tumor growth upon treatment with GRN163L for 50 days [21].

GRN163L has also been shown to be anti-metastatic in certain cancers. A549 lung cancer cells expressing luciferase (A549-luciferase) treated with GRN163L experienced a 50% reduction in rapid cellular attachment, and mouse xenografts models treated with GRN163L demonstrated a 53–92% reduction in tumor burden at 13, 20, and 27 days of treatment. It is suggested that these antiadhesive properties may be mediated by the lipid conjugation of the oligonucleotide with the palmitoyl group, the phosphorothioate backbone, and the guanine triplets of GRN163L [18]. These antiadhesive properties may also explain the anti-metastatic effects conferred by GRN163L. The same group showed that mouse tumor xenografts formed from A549-luciferase lung cancer cells pre-treated with GRN163L for three weeks had significantly reduced metastatic lesions compared to control cells [22]. Additionally, another study showed that treatment of A549 lung cancer cells with GRN163L resulted in reduced expression of E-cadherin, resulting in a disruption of cytoskeletal organization and loss of adhesive properties, which likely contributes to the anti-metastatic effects induced by GRN163L [23]. 

Despite numerous experimental studies showing its promising anticancer effects and its advancement to Phase II clinical trials, the application of GRN163L in clinical settings is limited largely due to its hematological toxicity and thus has not yet received FDA approval. A recent Phase II study utilized GRN163L as a therapy in children with recurrent CNS malignancies, specifically recurrent medulloblastoma, high-grade glioma, or ependymoma; it investigated the inhibition of telomerase and the responses to GRN163L treatment. Patients were given 285 mg/m^2^ of GRN163L 12–24 h before surgical resection of tumors and were additionally given IV GRN163L post-surgery on days 1 and 8 of a 21-day cycle. A total of 42 patients were enrolled, and though the evaluable patients experienced a 95% reduction in telomerase activity, no objective tumor responses were observed. Additionally, several grade ¾ toxicities were observed, and two patients died of intratumoral hemorrhage secondary to thrombocytopenia, which led to premature closure of the study [12]. Another Phase II study assessed the efficacy of GRN163L as a “switch” therapy in patients with advanced non-small cell lung cancer. Common grade ¾ toxicities included neutropenia and thrombocytopenia; however, no improvement in progression-free survival was observed in these patients after GRN163L treatment [16]. These toxicities that arise with GRN163L treatment require frequent drug holidays, which could allow telomere elongation and possible restoration of original telomere length, thus limiting the efficacy of GRN163L as a therapeutic [17].

Another concern with the use of GRN163L as a therapeutic is its effects on mesenchymal stem cells. Telomerase is expressed during embryonic development and is continually expressed by mesenchymal stem cells, and it is thus believed that GRN163L may have deleterious side-effects on the body’s stem cell population. Indeed, GRN163L has also been shown to alter the morphology of mesenchymal stem cells. Rat mesenchymal stem cells treated with GRN163L changed from a mesenchymal to a rounded morphology and lost adhesion to the cell culture surface, similar to the effects seen in cancer cells. Additionally, stem cells treated with GRN163L appeared to be arrested in the G1 phase of the cell cycle [24]. However, one week after removal of GRN163L treatment, normal mRNA and protein expression was again observed, as well as typical mesenchymal morphology. Thus, this suggests that GRN163L may not interfere with mesenchymal stem cell renewal and differentiation, at least in short-term treatment in vitro [24]. 

Though application as a stand-alone treatment is presently ineffective, GRN163L has shown promising anticancer effects as a combinatorial therapy. A recent study assessed the efficacy of GRN163L in combination with 3-aminobenzamide (3AB), a poly(ADP-ribose) polymerase inhibitor. Poly(ADP-ribose) polymerases catalyze the addition of poly(ADP-ribose) chains onto proteins of the shelterin complex (namely TRF1 and TRF2) and cause them to dissociate from the telomere, providing telomerase with access to the telomere. Pancreatic cancer cells with resistance to GRN163L treated with both GRN163L and 3AB demonstrated increased telomere shortening and decreased cellular lifespan. As poly(ADP-ribose) polymerase inhibitors are also frequently employed as anticancer therapeutics, this study suggests that the combination of these inhibitors with GRN163L may prove to be an effective therapy in cancers that overexpress telomerase, as well as those that are resistant to GRN163L [25]. Another study investigated the combination of GRN163L with trastuzumab, a monoclonal antibody against human epidermal growth factor receptor 2 (HER2), in HER2(+) breast cancer. HER2 overexpression is commonly associated with an increase in cancer stem cell (CSC) population, which are believed to play a role in driving tumor progression and metastasis. Both GRN163L alone and in combination with trastuzumab decreased CSC population and function. Furthermore, tumor growth in breast cancer tumor xenograft models was lower in combination therapy than either drug alone [26]. Additionally, GRN163L treatment has been shown to restore sensitivity of HER2(+) breast cancer to trastuzumab that have developed acquired trastuzumab resistance [27]. Other studies have shown that inhibition of telomerase sensitizes cancer cells to other anticancer agents [28,29]. In the past, it was rather unclear whether telomerase inhibition or the shortening of telomeres that results from telomerase inhibition was responsible for the sensitization to these drugs, as well as possibly responsible for the other anticancer effects induced upon telomerase inhibition. However, a study by Uziel et al. in 2010 showed that it was telomere shortening, and not telomerase inhibition per se, that increased sensitivity of Ewing sarcoma, breast carcinoma, and chronic myeloid leukemia to cisplatinum in a length-dependent manner. However, sensitivity to doxorubicin and vincristine was not increased significantly by telomere shortening [29]. These results suggest that the anticancer and synergistic effects of GRN163L may be due to subsequent telomere shortening, and not inhibition of telomerase in-and-of-itself.

In addition to combination with other molecularly-targeted therapies, GRN163L has shown efficacy in sensitizing cancer cells to conventional treatments, such as radiation therapy. A study using Ewing sarcoma, breast cancer, and chronic myelogenous leukemia cells showed that ionizing radiation treatment causes upregulation of telomerase activity in cancer cells via the PI3K/Akt pathway, and thus it reasons that inhibition of telomerase may enhance the cytotoxic effects of radiation therapy [30]. One study using esophageal cancer cells showed that cells treated with GRN163L underwent significantly higher levels of apoptosis than untreated cells after undergoing radiation therapy. Additionally, mouse esophageal cancer xenografts showed enhanced apoptosis and inhibition of proliferation following radiation treatment combined with GRN163L [31]. Another study in mouse orthotopic glioblastoma xenograft models also showed that after treatment with 30 mg/kg of GRN163L triweekly for a month followed by five days of radiation therapy, mice had an increased overall survival compared to those treated with radiation or GRN163L alone. Tumor growth was also decreased by 34% compared to either GRN163L or radiation therapy alone [32]. Further studies are certainly required to identify the optimal dosage and treatment period of GRN163L to enhance anticancer effects while limiting hematological toxicity. Additionally, studying telomerase inhibition in mice and relating this information to human cancer therapy is complicated by the fact that telomere and telomerase systems in murine models are radically different from humans. Murine telomeres are 5 to 10 times longer than human telomeres. Additionally, telomerase deficiency in humans results in severe conditions such as aplastic anemia, pulmonary fibrosis, and cirrhosis, while hematopoietic deficiency and development of emphysema after cigarette smoke exposure are among the only consequences of telomerase deficiency in mice [33]. However, these findings suggest that GRN163L may prove effective in combination therapies, and further studies are required to identify additional agents that may lead to greater clinical efficacy.

## 3. T-oligos

T-oligos, or telomere homolog oligonucleotides, have recently become an attractive research target for the development of targeted anticancer therapies. As their name suggests, these oligonucleotides are homologous to the 3′ overhang of mammalian chromosomes and have demonstrated significant anticancer activity in several cancer types, both in vivo and in vitro. Earlier studies by us and other investigators indicate T-oligo has minimal or no toxicity in mice, melanocytes or other normal cells [4,5,8,34,35]. In cancer cells, T-oligo targets abnormal regulatory signaling pathways including DDRs [4,36,37,38,39]. Upon accumulating in the nucleus after exogenous addition, T-oligos induce potent DNA damage responses (DDRs), such as cell cycle arrest and apoptosis, which may be mediated by the ataxia telangiectasia mutated (ATM) pathway and its downstream effectors p53, pRb, E2F1, cdk2, and p95/NBS1 in cancer cells [4,5,8,35,40]. The DDRs induced by T-oligo treatment are similar to those induced by telomere uncapping, as well as knockdown of the shelterin protein TRF2, which is responsible for the maintenance of telomere secondary structure [7,41,42]. 

**Figure 2:**
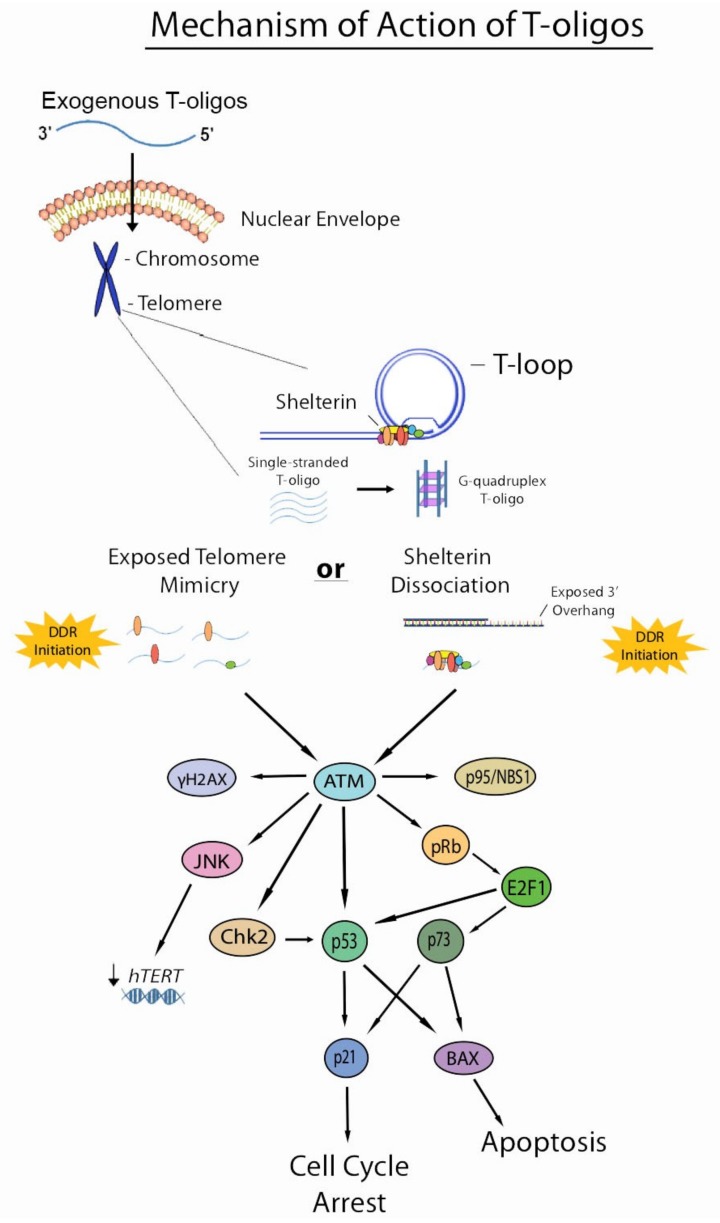
Current understanding of the mechanism of action of T-oligos. T-oligos accumulate in the nucleus and forms an intermolecular G-quadruplex structure. It is hypothesized that T-oligos then interact in some way with the shelterin complex, causing these proteins to dissociate from the telomere. DNA damage responses are then induced by either shelterin dissociation (as in the shelterin dissociation model) or mimicry of an exposed telomere (as in the telomere mimicry model), activating the ATM pathway and resulting in cell cycle arrest, apoptosis, and possibly reduced expression of hTERT through activation of the JNK pathway, which may be mediated by ATM activation.

T-oligo has shown anticancer activity in multiple cancer cell lines in vitro, such as melanoma, lymphoma, lung, breast, prostate, pancreatic, colorectal, and ovarian cancers [4,32,36,37,38,39,40,41]. Treatment of pancreatic cancer cells (Mia-PaCa 2 cell line) with T-oligo (16-mer) demonstrated substantial reduction in cell viability of about 74% within a 48-h period [43]. Another study using B-lymphoid cancer cells showed a significant S-phase cell cycle arrest within a 24–72 h period after T-oligo (16-mer) treatment, though normal B and T cells were spared. Upregulation of p53, as well as a dose-dependent increase in caspase-3 activity, were observed in these cells after T-oligo treatment, demonstrating activation of intrinsic cell death signaling secondary to DNA damage [34]. Activation of the p53 and pRb pathways has been shown to be required in telomere homolog oligonucleotide-induced senescence in human fibroblasts [40]. Our study using melanoma mouse xenografts showed that treatment with T-oligo (11-mer) reduced the number of metastases by about 90–95%, as well as reduced tumor volume by about 84–88% [38]. Recent work in our lab has shown a 75% reduction in cell viability in melanoma cells treated with T-oligo for 48 h [4]. Upregulation of the c-Jun *N*-terminal kinase (JNK), a known mediator of apoptotic signaling in response to cellular stress and DNA damage, was also observed in melanoma cells treated with T-oligo for a 24-h period [4]. Furthermore, T-oligo has been shown to activate the ATM pathway in melanoma and human breast carcinoma cell lines, inducing upregulation and phosphorylation of ATM and its downstream effectors p53, p73, p95/Nbs1, E2F1, p21, and BAX (Figure 2) [38,42].

T-oligo has also been shown to induce other processes associated with DNA-damage signaling. One study demonstrated that T-oligo treatment induced autophagy in malignant human glioma cells, while sparing normal astrocytes. Numerous autophagic vacuoles were present after a 72-h treatment with T-oligo with no evidence of apoptotic markers [44]. Other studies have shown that T-oligo treatment decreases angiogenic activity in non-small cell lung cancer and melanoma [36,45]. MM-AN melanoma cells treated with a 16-mer T-oligo demonstrated decreased VEGF, VEGFR2, angiopoietin-1 and-2 expression, as well as decreased secretion of VEGF. Activity of HIF-1α, a transcription factor that largely controls angiogenesis, was decreased, whereas activity of E2F1, a transcription factor involved in retinoblastoma-mediated apoptosis, was increased. Injection of T-oligo into melanoma mouse xenografts also reduced microvascular density and functional vessel density by 60% and 80%, respectively [45]. Our studies show that in non-small cell lung cancer xenograft models, H358 and SW1573 tumors treated with T-oligo (11-mer) had a 2.2-fold and 3.0-fold reduction in vessel density, respectively. VEGF staining was also decreased in these tumor models, further demonstrating the antiangiogenic activity of T-oligo [36]. 

Two main models generalize the mechanism by which T-oligos induce their anticancer effects in cancer cells. These models have been summarized succinctly by Ivancich, et al. The first model has been termed the “exposed telomere mimicry model” (ETM). This model hypothesizes that once T-oligo accumulates in the nucleus of a cancer cell, DNA damage-sensing proteins detect these oligonucleotides as damaged DNA due to their homology to the telomere. These proteins then initiate DDRs that mirror those induced under typical physiological instances of DNA damage. The second model is termed the “shelterin dissociation model” (SDM) that revolves around interactions of T-oligo with shelterin proteins. In this model, once T-oligo accumulates in the nucleus of a cancer cell, it interacts with the shelterin proteins, triggering their dissociation from the telomere. These proteins then bind to T-oligo and are sequestered from the telomere, thus exposing the telomere overhang and inducing DNA damage responses [46]. Though it is quite possible that exposed telomere mimicry is partially responsible for the DDRs induced by T-oligo treatment, recent evidence suggests that shelterin dissociation is also involved in the mechanistic action of T-oligo. A recent study using melanoma cells showed an upregulation of TRF2 by 2.2- and 3.0-fold and POT1 by 3.0-fold after T-oligo treatment for 48 and 72 h, respectively, by Western blotting. Upregulation was further verified by immunofluorescence, demonstrating an upregulation of 2.4- and 2.0-fold for TRF2 and POT1, respectively. Furthermore, binding of TRF2 and POT1 to T-oligo was verified by pull-down assay, suggesting that dissociation of shelterin proteins and their subsequent binding to T-oligo is indeed involved in the mechanism of action of T-oligo [4]. However, similar studies in colorectal cancer cells showed downregulation of these proteins upon T-oligo treatment, warranting the need to investigate these effects in other cancers [7]. Shelterin proteins may be degraded in certain cancers and rapidly synthesized in others to compensate for loss of these proteins. Further studies are required to understand the interaction of T-oligo with shelterin proteins and the molecular mechanism of DDR induction by T-oligo at the level of the telomere. 

Earlier studies suggest that telomerase activity is independent of T-oligo and thus modulated telomerase plays no role in either of the proposed models [36,40,46,47]. However, recent evidence indicates that the mechanism of action of T-oligo may indeed involve modulated expression of telomerase subunits and possibly telomerase activity. A recent study in our lab demonstrated that upon treatment of melanoma cells with T-oligo, expression of hTERT is reduced by 50%, which may suggest that a reduction in telomerase activity is associated with T-oligo treatment. Furthermore, it was also found that inhibition of JNK partially reversed this decrease in hTERT expression, which suggests that part of the mechanistic action of T-oligo may indeed involve reduced telomerase activity mediated by JNK [4]. Future directions should involve examining activity of telomerase activity directly, as several pre- and post-translational processes are largely involved in the regulation of telomerase protein levels and activity [30]. Further research is also required to investigate the role of modulation of telomerase activity in the anticancer responses of T-oligo. Additionally, though numerous studies have been done on cancers with ectopic expression of telomerase, little research has been devoted to examining the effects of T-oligo in cancers that perform alternative lengthening of telomeres (ALT). One study performed with the U20S osteosarcoma cell line, which is ALT-positive, examined the involvement of Werner syndrome helicase (WRN) in T-oligo-induced responses in these cells. WRN is an ATP-dependent helicase with exonuclease activity that, when mutated, leads to the development of Werner syndrome. This study showed that siRNA knockdown of WRN reduced phosphorylation of γH2AX, p53 and ATM in osteosarcoma cells after T-oligo treatment [48]. While this study suggests that T-oligo may induce some DDRs in ALT-positive cells, studies using other ALT-positive cells lines are also required to understand how T-oligo may induce these responses within these cells. 

While T-oligo has demonstrated significant anticancer activity in several cancer cell lines, both in vitro and in vivo, rapid degradation by nucleases remains a challenging obstacle in the application of T-oligo that limits its progression into clinical trial. In order to bolster the stability of T-oligo and improve delivery into the cell, our lab has recently investigated the efficacy of T-oligo complexed with an α-helical cationic peptide, PVBLG-8 (PVBLG). PVBLG readily complexes with oligonucleotides, thus neutralizing their negative charges and enhancing transfection. Furthermore, the helical structure of PVBLG confers enhanced stability across varying temperatures, pH, and salt concentrations. The T-oligo-PVBLG complex was shown to enhance cellular uptake by about 15-fold. Additionally, treatment of immunodeficient mice bearing melanoma xenograft tumors with T-oligo-PBVLG complexes demonstrated a 9-fold reduction in tumor volume, whereas T-oligo alone reduced tumor volume by only 3-fold. This complex was also shown to be effective in inhibiting angiogenesis in these mouse models [49]. While this complex has been shown to be effective as a delivery mechanism for T-oligo that enhances its anticancer activity, it currently has not been tested clinically. Another delivery system has also been studied using the cationic polymer, spermine. Star-shaped tetraspermine, formed by four spermine molecules with an EDTA core, was complexed with T-oligo and analyzed for cellular uptake and growth inhibition in prostate cancer cells. Uptake of this complex was markedly greater than of T-oligo alone. Furthermore, the star-shaped tetraspermine-T-oligo complex induced cytotoxic effects in the cells at concentrations 10–20-fold lower than of T-oligo alone [8]. These findings suggest that complexing T-oligo with cationic macromolecules may prove to be an effective method of delivery. However, further studies are required to investigate other delivery options that are effective, low-cost, and confer minimal cytotoxicity to normal cells.

Recent data has shown that, due to its guanine-rich nature, T-oligo is able to adopt a four-stranded intermolecular G-quadruplex structure formed by the hydrogen bonding of guanine residues in a lariat formation, similar to those that form endogenously in the D-loop of the telomere. Studies have shown that this G-quadruplex structure maintains greater stability than single-stranded T-oligo and similar cellular uptake. Interestingly, in a nuclease digestion assay, it was found that G-quadruplexes first degrade into the single-stranded T-oligos before complete digestion. These G-quadruplexes also maintained antiproliferative activity in melanoma cells, albeit reduced compared to single-stranded T-oligo [4]. These findings may suggest that, given their enhanced stability, these T-oligo G-quadruplexes may prove to be an improved therapy in vivo whereas single-stranded T-oligos are highly susceptible to degradation by nucleases. Data also suggests, however, that T-oligo may localize to the nucleus of cancer cells and form G-quadruplexes within the nucleus, and it is currently unclear whether or not these intracellularly-formed T-oligo G-quadruplexes are requisite in the mechanistic action of T-oligo [4]. This is further complicated by the fact that endogenous G-quadruplexes and T-oligo G-quadruplexes are sequentially identical and difficult to distinguish within the cell. Further studies are certainly required to elucidate the involvement of these G-quadruplexes in T-oligo-induced DDRs and how they may interact with telomere architecture or shelterin proteins.

The application of T-oligo in combinatorial therapies has also yielded promising results. One study showed that combinatory treatment of non-small cell lung cancer cells with T-oligo and the EGFR inhibitor Gefitinib, which is widely used clinically, demonstrated an additive inhibition of cell growth that was significantly greater than Gefitinib alone [7]. T-oligo has been shown to resensitize human ovarian cancer cells to tumor necrosis factor-related apoptosis-inducing ligand (TRAIL) by inducing expression of the TRAIL receptors in the cells [50]. Additionally, T-oligo has been shown to increase sensitivity of mammary carcinoma cells to radiation treatment, both in vivo and in vitro, synergistically enhancing efficacy of radiation therapy [51]. These findings suggest that T-oligo may prove to be an effective anticancer agent in combination with established therapies if not applied as an individual therapeutic.

## 4. miRNAs Targeting Telomerase

MicroRNAs (miRNAs) are endogenous non-coding RNAs involved in post-transcriptional gene expression regulation by complexing with the RNA-induced silencing complex (RISC) and base pairing with a target mRNA, generally preventing translation of the mRNA or initiating its cleavage by RISC, effectively silencing the associated gene. It is predicted that over 60% of all protein-coding genes are partially regulated via miRNA-induced silencing [9]. Recently, miRNAs have garnered interest in the development of cancer therapeutics due to their involvement in driving carcinogenesis or their ability to silence genes associated with carcinogenesis. Additionally, given their presence in tissues and the bloodstream, they are attractive cancer biomarkers. Generally, miRNAs that play a role in carcinogenesis are divided into two groups: oncogenic miRNAs, which promote cancer development, and tumor suppressor miRNAs, which inhibit cancer development [9,52,53]. Since telomerase activation results in unlimited replicative potential in over 90% of cancers, several miRNAs that regulate expression of telomerase subunits at the post-transcriptional level have been studied. Several miRNAs, such as MiR-138, MiR-128, MiR-1182, MiR-133a, MiR-342, MiR-491, and MiR-541, have been shown to negatively regulate expression of hTERT via interaction with hTERT mRNA, acting as tumor suppressor miRNAs [9,54]. In cervical cancer cells, MiR-138 overexpression has been shown to inhibit cell proliferation, migration, invasion, induce apoptosis, and also inhibit cervical cancer tumor growth in vivo [10]. Downregulation of MiR-138 has been shown to be associated with an overexpression of hTERT in human anaplastic thyroid carcinoma cells [55]. Additionally, overexpression of MiR-138 was shown to be more effective than shRNA-mediated knockdown of hTERT in potentiating flavonoid Apigenin-induced apoptosis in neuroblastoma cells [56]. Additionally, another study demonstrated that MiR-1182 reduced gastric cancer cell proliferation and migration in vitro and in vivo by targeting the open reading frame (ORF) of hTERT mRNA [57]. Altered expression of MiR-128 has been found in several cancer types, such as osteosarcoma, glioma, and acute lymphoblastic leukemia. MiR-128 has been shown to act as both an oncogenic miRNA as well as a tumor suppressor miRNA, as it binds to multiple targets [9,58,59]. A recent study showed that MiR-128 is able to bind to the coding sequence of hTERT mRNA, and overexpression of this miRNA reduced hTERT mRNA as well as TERT protein levels in HeLa cells. Binding of MiR-128 to hTERT mRNA prevents translation of the catalytic subunit of telomerase, thus likely reducing telomerase activity. Additionally, it was found that MiR-128 is also able to bind to the mRNA sequence of the reverse transcriptase component of retrotransposons (LINE-1), supporting the notion that this miRNA and other miRNAs may be able to regulate cellular processes by interaction with the mRNA of multiple reverse transcriptases and other enzymes [9].

MiRNAs targeting hTERT mRNA may act essentially as telomerase inhibitors in clinical applications, and a combination of suppressor miRNAs with currently available chemotherapy drugs is an approach that has garnered a great deal of interest [60]. However, inhibition of telomerase as a clinical approach is complicated by the lengthy timeframe before inactivation of telomerase leads to critical telomere shortening. In the case of GRN13L, studies have shown that telomerase inhibition induces anticancer effects in cancer cells after weeks of treatment [19,21]. Additionally, the effects of miRNAs that reduce telomerase activity in stem cells has not yet been explored and requires further study. Furthermore, evidence suggests that telomerase localizes to the mitochondria under stress, and thus long-term telomerase inhibition may have unforeseen consequence [61,62]. Additionally, inhibition of telomerase causing progressive telomere shortening may result in telomere crisis and dysfunction events that may increase genomic instability and enhance cancer aggression [61]. Future studies of these miRNAs and other telomerase-inhibiting strategies should certainly consider these pitfalls and how to overcome them. However, miRNAs have also shown to be valuable biomarkers in tumors and blood in multiple cancers, such as prostate, lung, and breast cancer [63,64,65]. For example, a 34 miRNA panel was recently developed for high-risk smokers to identify early stages of lung cancer [64]. Expression of hTERT is likewise an important prognostic marker for several cancers [66,67,68]. Therefore, analysis of circulating oncogenic or tumor suppressor miRNAs that target hTERT expression could be evaluated as a useful diagnostic/prognostic tool.

Several studies have also identified multiple oncogenic miRNAs that drive oncogenic transformation and cancer aggression by upregulating hTERT. One such miRNA is MiR-21, which has been implicated in several oncogenic processes in malignant melanoma, such as evasion of apoptosis, enhanced invasiveness, genetic instability, oxidative stress, and proliferation [69]. It has also been shown that MiR-21 is overexpressed by 3.4-fold in colorectal cancer and hTERT is subsequently overexpressed by 2-fold [70]. It is also suggested that MiR-21 increases expression of hTERT via several pathways. MiR-21 has been shown to upregulate hTERT expression in hypertrophic scar fibroblasts via targeting of the 3′-UTR of phosphatase and tensin homologue (PTEN). PTEN acts as an inhibitor of Akt, thus preventing downstream proliferative signaling. Transfection of a MiR-21 mimic in hypertrophic scar fibroblasts caused an upregulation of hTERT at both the protein and mRNA level, as well as increased PI3K/Akt signaling. Furthermore, overexpression of PTEN in these cells inhibited the upregulation of hTERT and PI3K/Akt signaling associated with MiR-21 overexpression [71]. Another study found that MiR-21 plays a major role in carcinogenesis of glioblastoma mediated through STAT3, a transcription factor largely involved in the differentiation of TH17 helper T cells. Knockdown of MiR-21 in glioblastoma mouse xenografts showed marked reduction in tumor growth as well as reduction of hTERT and STAT3 expression. Several other oncogenic miRNAs have been identified that drive carcinogenesis by upregulation of telomerase, such as MiR-19b and MiR-346 [72,73]. MiR-346 has also been shown to compete with MiR-138 (which reduces hTERT expression) for the 3′-UTR of hTERT mRNA, thus competitively regulating hTERT expression [72]. 

Targeting these oncogenic miRNAs may be an effective route of therapy for cancers with high expression of hTERT. A common approach to target these miRNAs is the use of antimiRNAs, which are antisense to the target miRNA and effectively block their action [74]. Because multiple miRNAs are often upregulated together in cancer, in miRNA “families”, an antimiRNA approach using miRNA “sponges” is often used to target multiple miRNAs that are upregulated together. These “sponges” are DNA with artificial miRNA binding sites located in the 3′-UTR of a gene [74]. A recent study showed that the use of synthetic miRNA sponges using miRNA binding sites driven by TERT promoter mutations inhibited the progression of bladder cancer by binding and inhibiting the activity of four oncogenic miRNAs [75]. Some miRNAs have also been targeted using small molecule inhibitors. Pre-miRNA possesses a druggable narrow groove that can be bound with small positively-charged RNA specific ligands, inhibiting the respective miRNA [74,76]. Researchers have developed multiple platforms to screen interaction of small molecules with miRNAs. One example is Inforna, a platform used to design small molecules that interact with specific RNAs based on sequence [77]. Additionally, knockdown of genes coding for these miRNAs using CRISPR/Cas9 has also been explored by several groups [78,79,80]. 

The application of miRNA inhibition as a therapy is underway for several diseases, though few have progressed to clinical trials. Inhibition of miR-122, which prevents replication of the hepatitis C virus, is currently the farthest along in Phase II clinical trial [74,81]. MiRNA inhibition for cancer therapy remains largely in preclinical stages [74]. However, MRG-106 is an oligonucleotide antimiRNA of miR-155 and is currently undergoing Phase I clinical trials. A recent Phase I study using MRG-106 showed that cutaneous T-cell lymphoma patients demonstrated improvement in either individual lesions or total skin disease over the course of the study, prompting application of this therapy to other malignancies [82]. Studies inhibiting oncogenic miRNAs that enhance telomerase activity has not yet progressed to clinical trials, but further studies targeting these miRNAs using in vivo models warrant further investigation of this therapeutic approach for the development of clinical applications. 

## 5. Conclusions

Molecularly-targeted therapies are an extremely promising approach in the treatment of aggressive cancers where conventional cancer therapies are lacking. Additionally, the deleterious side-effects associated with conventional therapies can largely be bypassed through the use of molecularly-targeted therapies due to their specificity [83]. Telomeres and telomerase remain attractive targets for the development of novel moleculary-targeted therapies in order to eliminate the unlimited replicative potential that is characteristic of many cancers. The therapies discussed in this review have been shown to be effective anticancer agents. However, further research is warranted to fully elucidate the effects these therapies have on cancers in vitro and in vivo, as well as to optimize their application in clinical settings. 

While inhibition of telomerase is an efficacious strategy to treat cancers, and the expression of its catalytic subunit hTERT is a useful prognostic marker in many cancers [66,67,68], the majority of current studies have found clinically relevant doses of GRN163L as a stand-alone therapy to be too toxic [12,16,17]. Potentially, telomerase inhibition could stand as a beneficial addition to other treatment methods, whether they be molecularly-targeted or conventional therapies, when administered at lower, non-toxic doses. Further study of GRN163L may also uncover other therapeutic effects beyond inhibiting proliferation and metastasis, such as reducing angiogenesis or immune system involvement. Additionally, though short-term treatment of stem cells with GRN163L in vitro has shown reversible effects [24], the long-term effects of GRN163L treatment on stem cells has not yet been explored and requires further investigation. 

Despite the substantial evidence of their anticancer effects [34,38,43] T-oligos have not yet progressed to clinical trial. This delay is largely attributed to the incomplete elucidation of its mechanism of action and difficulty in its delivery. Though DDRs induced by T-oligos have been shown to largely center around the ATM pathway [38,40,42], further studies are needed to investigate the preliminary events initiating these responses, as well as what other processes affect downstream targets of this signaling cascade. While evidence does seem to support the shelterin dissociation model of DDR induction, data regarding the effects on the expression of shelterin proteins seems to differ across various cancers, warranting the continued investigation of T-oligo’s interaction with shelterin components in several cancers [4,7,46]. Additionally, further studies are required to understand the role of endogenous T-oligo G-quadruplex formation in the mechanism of action of T-oligos. Previous studies have also suggested that the mechanism of T-oligo-induced DDRs is independent of telomerase [40]. However, recent evidence also seems to contradict this, demonstrating, at least in melanoma, that telomerase may indeed be modulated by T-oligo [4]. Though several studies have shown that T-oligo has minimal to no effects on normal cells, the mechanism behind this cancer specificity is largely unexplored [34,36,44]. Perhaps the presence of telomerase activity or lack of regular cell cycle checkpoints in cancer cells may in some way be part of its mechanism. 

There has been an explosion in research interest on the use of miRNAs, whether they be suppressor or oncogenic, in cancer therapy due to their involvement in tumorigenesis and cancer progression. However, the application of these miRNAs is complicated by several challenges. MiRNAs have limited stability in biological systems and are susceptible to degradation by nucleases. Additionally, exogenous addition of certain miRNAs may activate sequence-specific immune responses that may interfere with the activity of these miRNAs [84,85]. Further development of delivery mechanisms and modifications to enhance miRNA stability are required to apply miRNAs as future therapies. Examples of such modifications include nucleic acid locking (locking of the 3’ *endo* moiety of ribose), glycation, and backbone modification [86,87]. Furthermore, since miRNAs are secreted by cells to exert effects on the cellular microenvironment, the exogenous addition of miRNAs may have off-target effects on cells [60]. Thus, further studies are required to optimize these therapies in a variety of cancers. Continued research on oligonucleotide-based therapies that target telomeres and telomerase will certainly bolster current therapeutic interventions and improve cancer patient prognosis.
